# A High-Quality, Chromosome-Level Genome Provides Insights Into Determinate Flowering Time and Color of Cotton Rose (*Hibiscus mutabilis*)

**DOI:** 10.3389/fpls.2022.818206

**Published:** 2022-02-14

**Authors:** Yuanzhao Yang, Xiaodan Liu, Xiaoqing Shi, Jiao Ma, Xinmei Zeng, Zhangshun Zhu, Fangwen Li, Mengyan Zhou, Xiaodan Guo, Xiaoli Liu

**Affiliations:** ^1^Chengdu Botanical Garden, Chengdu, China; ^2^Novogene Bioinformatics Institute, Beijing, China

**Keywords:** *Hibiscus mutabilis*, genome, Hi-C, phylogenetic affiliation, floral regulators

## Abstract

*Hibiscus mutabilis* (cotton rose) is a deciduous shrub or small tree of the Malvaceae family. Here, we report a chromosome-scale assembly of the *H. mutabilis* genome based on a combination of single-molecule sequencing and Hi-C technology. We obtained an optimized assembly of 2.68 Gb with a scaffold N50 length of 54.7 Mb. An integrated strategy of homology-based, *de novo*, and transcriptome-based gene predictions identified 118,222 protein-coding genes. Repetitive DNA sequences made up 58.55% of the genome, and LTR retrotransposons were the most common repetitive sequence type, accounting for 53.15% of the genome. Through the use of Hi-C data, we constructed a chromosome-scale assembly in which Nanopore scaffolds were assembled into 46 pseudomolecule sequences. We identified important genes involved in anthocyanin biosynthesis and documented copy number variation in floral regulators. Phylogenetic analysis indicated that *H. mutabilis* was closely related to *H. syriacus*, from which it diverged approximately 15.3 million years ago. The availability of cotton rose genome data increases our understanding of the species’ genetic evolution and will support further biological research and breeding in cotton rose, as well as other Malvaceae species.

## Introduction

*Hibiscus mutabilis* is one of the most popular tree species in the Malvaceae family, which includes species such as *Gossypium raimondii* and *Hibiscus syriacus* (Rose of Sharon). Some members of the Malvaceae have relatively high economic value. For example, cotton is the largest source of natural textile fibers in the world, and over 90% of its annual fiber production comes from allotetraploid cotton (*G. hirsutum* and *G. barbadense*) (Wang et al., 2019). Additionally, many Malvaceae species are used as ornamentals because of their flowers. *H. syriacus* is an important horticultural species whose attractive white, pink, red, lavender, or purple flowers are displayed over a long bloom period, although individual flowers last only a day in the landscape (Kim et al., 2016). This study of *H. mutabilis* (2*n* = 92) (Li et al., 2015) focuses primarily on its ornamental characters, including its flower colors, long bloom time, and floral development and morphogenesis. In addition to its ornamental value, *H. mutabilis* is also an ingredient in local herbal remedies. It is thought to cool the blood, relieve toxins, reduce swelling, and alleviate pain, and it has long been used in the treatment of ulcers, swelling, herpes zoster, scalding, bruises, etc. (Liu et al., 2015). The complete genome sequence of an organism provides a large amount of information for subsequent biological studies (Yang et al., 2005). The *H. mutabilis* genome sequencing project is therefore extremely valuable for breeding, comparative genomics research, and other activities.

**FIGURE 1:**
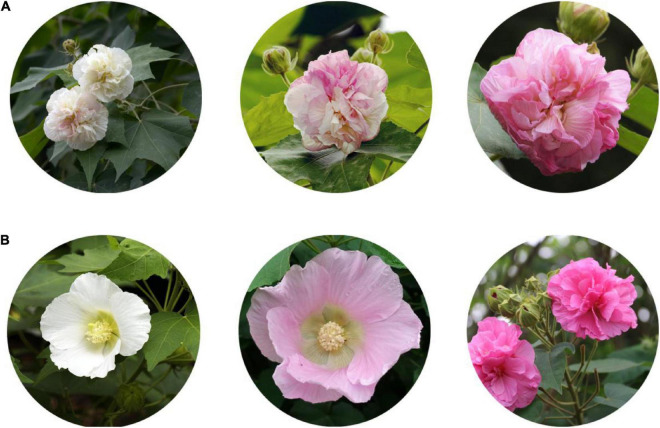
*Hibiscus mutabilis* floral morphology. **(A)** Dynamic change in the flower color of *H. mutabilis* f. *mutabilis* ‘Drunk girl’ from white to pink. **(B)** A variety of colors found in different color cultivars of *H. mutabilis*, and from left to right in turn are *H. mutabilis* ‘Single-Petal Pink,’ *H. mutabilis* ‘Single-Petal White’ and *H. mutabilis* ‘Purple silk.’

*H. mutabilis* has been cultivated for more than 2,000 years south of the Yangtse River; it is also the city flower of Chengdu and has great significance for the city. Commonly used as an ornamental species, its attractive purple, red, pink, or white flowers are displayed over a long bloom period (3–4 months or more), although its individual flowers last for less than 48 h (Figure 1). During flower development, the floral color of some varieties shows little change, but that of other varieties undergoes a marked change from white to pink within a single day. This interesting dynamic phenomenon can be seen in the cultivars ‘Drunk girl’ and ‘Bairihuacai’ and occurs during the process of individual flower development, unlike the color differences found in distinct cultivars of chrysanthemum (Ohmiya et al., 2006), *Narcissus pseudonarcissus* (Li et al., 2018), or *Brassica napus* (Zhang et al., 2015).

The development of genomic resources and molecular breeding technologies holds promise for targeted character improvement of *H. mutabilis* in the near future. Recently, a 1.75 Gb draft genome of *H. syriacus* was assembled, and a chromosome-scale genome of *H. cannabinus* was published in 2020 (Zhang et al., 2020). Many breeding systems and novel varieties have been produced using traditional methods to meet horticultural requirements, but a completed genomic sequence will accelerate the breeding of cotton rose.

**FIGURE 2:**
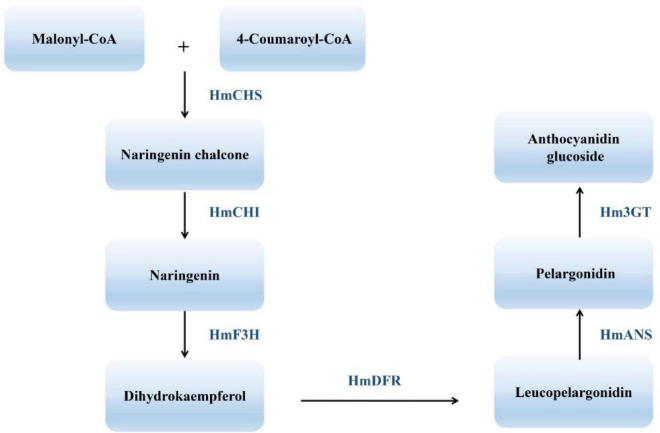
An abbreviated diagram of the flavonoid pathway that produces anthocyanins. CHS, chalcone synthase; CHI, chalcone isomerase; F3H, flavanone 3-hydroxylase; DFR, dihydroflavonol 4-reductase; ANS, anthocyanidin synthase; 3GT, 3-O-glucosyl transferase.

The many flower colors of cotton rose give it high ornamental value and reflect the complexity of the underlying flavonoid metabolic pathway. One endpoint of flavonoid biosynthesis is the production of anthocyanins, pigments that produce the colors of many flowers, fruits, and other plant tissues (Koes et al., 1994). Chalcone synthase (CHS), chalcone isomerase (CHI), flavanone 3-hydroxylase (F3H), dihydroflavonol reductase (DFR), anthocyanidin synthase (ANS), and flavonoid 3-O-glucosyltransferase (UFGT) all function in the synthesis of anthocyanins and anthocyanidins, their aglycone counterparts. CHS represents the first committed step in the flavonoid pathway (Meer et al., 1993). The second step is performed by CHI, which acts on the yellow naringenin chalcone product of CHS, catalyzing its isomerization to the colorless flavanone naringenin (Moustafa and Wong, 1967). Dihydroflavonols are subsequently reduced to leucoanthocyanidins by DFR (Durbin et al., 2004). ANS catalyzes the formation of cyanidin from leucoanthocyanidin and is the penultimate step in the biosynthesis of the anthocyanin class of flavonoids (Figure 2). Despite recent progress in understanding *H. mutabilis* anthocyanidin biosynthesis, the lack of a genome sequence has hampered efforts to elucidate the molecular and genetic determinants of this trait, which underlies the dynamic phenomenon of flower color development. Genome and transcriptome sequences are needed in order to fully analyze the molecular mechanisms of anthocyanidin biosynthesis.

In the present study, we generated a reference genome for *H. mutabilis* using a combination of single-molecule sequencing and Hi-C technology. We identified functional genes involved in the biosynthesis of anthocyanins based on homology searches and functional annotations. We also investigated copy number variation in floral regulators among multiple species to gain insight into the evolution of flowering phenotypes in *H. mutabilis*. The genomic resources developed here will be useful for further experimentation, cultivation, and breeding of *H. mutabilis* and other Malvaceae species.

## Materials and Methods

### Plant Materials and Whole-Genome Sequencing

The *H. mutabilis* material sequenced in this study was the stably heritable single-petal white color cultivars, which is cultivated in the nursery of the Chengdu Botanical Garden (CDBG), Sichuan, China. The breeding system of *H. mutabilis* belongs to allogamy. Seeds of ‘single-petal white’ were collected in the laboratory of the CDBG. Young leaves (∼3 cm width) were harvested to extract high-quality DNA for Illumina and Oxford Nanopore Technology (ONT) sequencing. For transcriptome sequencing, petals were manually collected from three color cultivars (‘single-petal white,’ ‘single-petal pink,’ and ‘Purple silk’) and at three stages of color development in ‘Drunk girl’ (white, blended white and pink, and fully pink). Flowers at the same stage from individual *H. mutabilis* plants were pooled and divided into three samples. These samples were immediately frozen in liquid nitrogen and then used for RNA sequencing.

High-quality *H. mutabilis* genomic DNA was extracted from young leaves with a DNA secure Plant Kit (TIANGEN, China) and used to construct long-read libraries for the ONT platform.^1^ Libraries were prepared following the ONT’1D Genomic DNA by Ligation (Kit 9 chemistry)-PromethION’ protocol and sequenced using the PromethION protocol. In addition, high-quality DNA was broken into random fragments, and an Illumina paired-end library was constructed with an insert size of 350 bp and sequenced using the Illumina HiSeq X Ten platform.

For Hi-C sequencing, leaves were fixed with 1% formaldehyde solution in MS buffer (10 mM potassium phosphate, pH 7.0; 50 mM NaCl; 0.1 M sucrose) at room temperature for 30 min in a vacuum. After fixation, the leaves were incubated at room temperature for 5 min under a vacuum in MC buffer with 0.15M glycine. Approximately 2 g of fixed tissue was homogenized with liquid nitrogen, resuspended in nuclei isolation buffer, and filtered with a 40-nm cell strainer. The procedures for enriching nuclei from flow through and subsequent denaturation were performed according to a 3C protocol. The chromatin extraction procedures were similar to those described previously. In brief, chromatin was digested for 16 h with 400 U *Hin*dIII restriction enzyme (NEB) at 37°C. DNA ends were labeled with biotin and incubated at 37°C for 45 min, and the enzyme was inactivated with 20% SDS solution. DNA ligation was performed by the addition of T4 DNA ligase (NEB) and incubation at 16°C for 4–6 h. After ligation, proteinase K was added for reverse cross-linking during overnight incubation at 65°C. DNA fragments were purified and dissolved in 86 μL of water, and unligated ends were then removed. Purified DNA was fragmented to a size of 300–500 bp, and DNA ends were repaired. Finally, DNA fragments labeled with biotin were separated on Dynabeads M-280 Streptavidin (Life Technologies). Hi-C libraries were assessed for quality and sequenced on an Illumina HiSeq X Ten sequencer.

### Genome Assembly and Chromatin Interaction Analysis Using Hi-C Technology

*De novo* assembly of all Nanopore long reads was performed using wtdbg2 v2.5 (Ruan and Li, 2020). Because Nanopore reads contain systematic errors in homopolymeric regions, we polished the consensus assembly three times using the Nanopore reads as input to Racon v1.3.1 (Vaser et al., 2017) and then three additional times using Illumina reads as input to Pilon v1.22 (Walker et al., 2014).

The Hi-C data were mapped to the original scaffold genome using BWA v0.7.7 (Li, 2009) and only reads with unique alignment positions were extracted to construct a chromosome-scale assembly using LACHESIS v201701 (Burton et al., 2013).

We used both CEGMA (Core Eukaryotic Gene Mapping Approach) (Parra et al., 2007; Supplementary Table 4) and BUSCO (Benchmarking Universal Single-Copy Orthologs) (Simão et al., 2015; Supplementary Table 5) to evaluate the completeness of the assembly.

### Genome Annotation

TEs were identified in the genome assembly at both the DNA and protein levels. We used RepeatModeler, RepeatScout (Tarailo-Graovac and Chen, 2009), Piler (Edgar and Myers, 2005), and LTR_FINDER (Xu and Wang, 2007) to develop a *de novo* TE library. RepeatMasker (Tarailo-Graovac and Chen, 2009) was used for DNA-level identification with Repbase and the *de novo* TE library. Tandem repeats were identified using Tandem Repeats Finder (Benson, 1999). At the protein level, RepeatProteinMask (Tarailo-Graovac and Chen, 2009) was used to conduct WU-BLASTX searches against the TE protein database. Overlapping TEs that belonged to the same type of repeat were integrated together.

We used homology-based, *de novo*, and transcriptome-based approaches to predict protein-coding genes in the *H. mutabilis* genome. For homolog-based prediction, sequences of homologous proteins from six plants (*A. thaliana, C. capsularis, D. zibethinus, G. raimondii, H. umbratica, H. syriacus*, and *T. cacao*) were downloaded from Ensembl, NCBI, or JGI. Protein sequences were aligned to the genome using TBLASTN with an *E*-value cutoff of 1 × 10^–5^. The blast hits were concatenated using solar (Yu et al., 2007). For each blast hit, GeneWise v2.4.1 (Birney and Clamp, 2004) was used to predict the exact gene structure in the corresponding genomic regions. The five *ab initio* gene prediction programs AUGUSTUS v3.0.2 (Stanke and Morgenstern, 2005), Genescan v1.0 (Aggarwal and Ramaswamy, 2002), GeneID (Parra et al., 2000), GlimmerHMM v3.0.2 (Majoros et al., 2004), and SNAP (Korf, 2004) were used for *de novo* protein prediction. To further optimize the genome annotation, RNA-seq data from floral, leaf, and stem tissues were aligned to the *H. mutabilis* genome using TopHat v2.0.13 (Trapnell et al., 2009) to identify exon regions and splice junctions. The alignment results were then used as input for Cufflinks v2.1.1 (Trapnell et al., 2010) in order to assemble transcripts into gene models. Trinity (Grabherr et al., 2011) was used with default parameters to assemble the RNA-seq data, and PASA (Haas et al., 2003) was used to improve the gene structures. A weighted and non-redundant gene set was generated by EVidenceModeler (EVM) (Haas et al., 2008), which merged all gene models predicted using the three approaches above. PASA adjusted the gene models generated by EVM based on information from the transcriptome assembly.

The functional annotation of protein-coding genes was evaluated by BLASTP (*E*-value ≤ 1 × 10-5) against two integrated protein sequence databases, Swiss-Prot (Bairoch and Apweiler, 2000) and the NCBI non-redundant (NR) database. Protein domains were annotated using InterProScan v4.8 to search InterPro v32.0 (Mulder and Apweiler, 2007), which includes the Pfam, PRINTS, PROSITE, ProDom, and SMART databases. Gene Ontology (GO) (Ashburner et al., 2000) terms for each gene were obtained from the corresponding InterPro descriptions. Putative pathway assignments for each gene were obtained by blasting against the KEGG (Kanehisa and Goto, 2006) database with an *E*-value cutoff of 1 × 10^–5^.

tRNA genes were predicted by tRNAscan-SE (Lowe and Eddy, 1997), and miRNA and snRNA fragments were identified using Infernal (Nawrocki et al., 2009) with the Rfam (Griffiths-Jones et al., 2005) database. rRNA genes were identified using BLASTN (*E*-value ≤ 1 × 10^–10^) against the plant rRNA database.

### Genome Evolution Analysis

First, nucleotide and protein data from nine species (*A. trichopoda, A. thaliana, B. ceiba, C. capsularis, D. zibethinus, G. raimondii, H. syriacus, R. chinensis*, and *T. cacao*) were downloaded from Ensembl, NCBI, and JGI. The longest transcript was selected from the alternatively spliced transcripts of each gene, and genes with ≤ 50 amino acids were removed. Nucleotide and protein data from *H. mutabilis* and the other nine angiosperms were clustered into orthologous groups using BLASTP and OrthoMCL v2.0.9, and an MCL inflation of 1.5 was used as the cluster granularity setting (Li et al., 2003). A phylogenetic tree was constructed using shared single-copy orthologs. Protein sequences of the orthologs were aligned using MUSCLE (Edgar, 2004), and the protein alignments were transformed to CDS alignments. We then concatenated the CDS alignments into a “supermatrix” from which the phylogenetic tree was constructed using the maximum likelihood (ML) TREE algorithm in RAxML v8.1.13 (Stamatakis, 2006) with the best-scoring protein substitution model (GTRGAMMA) and 1,000 bootstrap replicates. The MCMCtree program in the PAML package (Yang, 1997) was used to estimate divergence times among the ten species. Three fossil calibration points were used for restraining the age of the nodes: 23–48 Mya (Million years ago) for the MRCA of *T. cacao*–*G. raimondii*, 65–107 Mya for the MRCA of *G. raimondii*–*A. thaliana* (Wang et al., 2012), and 103–109 Mya for the MRCA of Malvales–Rosales (Wikström et al., 2001). CAFE was used to identify expansions and contractions within orthologous gene families by comparing cluster size differences between the ancestor and other species (De Bie et al., 2006). To estimate the synonymous substitutions per synonymous site (Ks), all paralogous gene pairs were analyzed with the ML method in PAML (Yang, 1997). MCscan (Tang et al., 2008) was used to analyze genome collinearity in *H. mutabilis*.

### Identification of Nucleotide-Binding Site-Encoding Genes

To identify NBS-encoding genes, representative genes from each plant genome were screened using a raw Hidden Markov Model (HMM3.0) (Marchin et al., 2005) to search for the Pfam NBS family PF00931 domain with an *E*-value cut-off of 1.0. All putative NBS protein sequences were analyzed and manually curated based on a TBLASTN search against known R gene sequences in GenBank. To further identify TIR homologs and sequences that encoded CC and LRR motifs, candidate NBS-LRR protein sequences were characterized using SMART (Schultz et al., 1998), the Pfam database (Finn et al., 2013), and the COILS program (Lupas et al., 1991) with a threshold of 0.9 to specifically detect the CC domain.

### Transcriptome Sequencing

For analysis of flowering gene(s), petals were manually collected from three color cultivars at the same time (2–3 p.m.) and at three stages of color development in ‘Drunk girl’ (white at the 9 a.m., blended white and pink at the 12 a.m., and fully pink at 6 p.m.) and these samples were immediately frozen in liquid nitrogen and then used for RNA sequencing, and total RNA was extracted using an RNAprep Pure Plant Kit (TIANGEN, China). The quality and quantity of the RNA samples were evaluated using a NanoPhotometer (Implen, CA, United States), a Qubit 3.0 Fluorometer (Thermo Fisher Scientific, United States), and an Agilent 2,100 Bioanalyzer (Agilent Technologies, United States). All RNA samples with integrity values greater than 7.0 were used for cDNA library construction and sequencing. The cDNA libraries were prepared using the NEB Next Ultra RNA Library Prep Kit (E7350L, NEB, United States), and 150-bp paired-end sequencing was performed on the Illumina NovaSeq 6000 platform (Illumina, CA, United States).

## Results

### Genome Sequencing and Assembly

**FIGURE 3:**
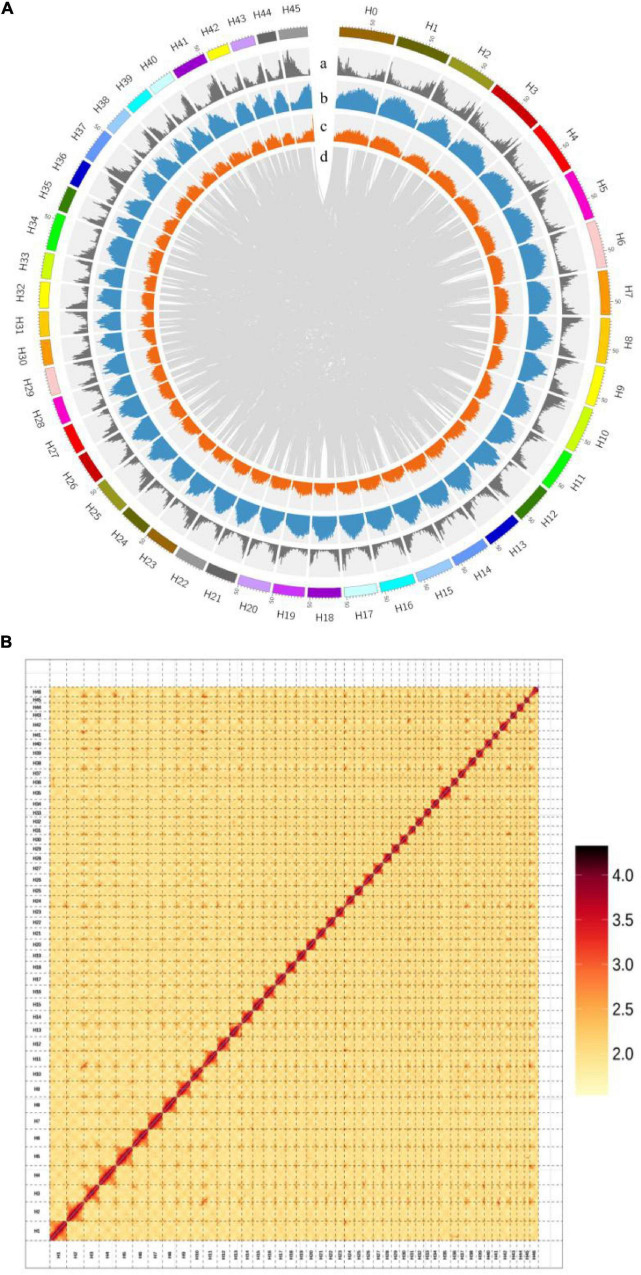
**(A)** Chromosomal features of *H. mutabilis*. (a) Gene density; (b) Repeat density; (c) AT content; (d) Syntonic blocks. **(B)** Hi-C map of the *H. mutabilis* genome showing genome-wide all-by-all interactions. The map shows a high resolution of individual chromosomes that were scaffolded and assembled independently. Color intensity indicates the frequency of Hi-C interaction links from low (yellow) to high (red).

We assembled the *H. mutabilis* genome using a combination of Illumina HiSeq X Ten and Oxford Nanopore PromethION sequencing. We generated 315.22 Gb (104-fold coverage) of raw 150-bp paired-end Illumina reads and 469.91 Gb (155-fold coverage) of raw Nanopore reads. The genome size was estimated to be 3032.98 Mb based on the 17-mer depth distribution (Supplementary Table 1 and Supplementary Figure 1). Nanopore long reads were assembled into contigs and scaffolds using wtdbg2 v2.5,13 resulting in a final assembly of 2.68 Gb with 5,464 contigs and a contig N50 of 2.22 Mb (Supplementary Table 2). Its GC percentage was 35.36%, similar to that of the *H. syriacus* genome (34.04%). In total, 363.5 Gb of clean reads were obtained from Hi-C sequencing (over 121-fold coverage). We used these data to construct chromosome-scale scaffolds, resulting in a total of 5,598 contigs, with a scaffold N50 of 54.70 Mb and a total length of 2,676,237,573 bp (Supplementary Table 10 and Figure 3A).

Next, The clustering of contig by hierarchical clustering of the Hi-C data was performed. Hi-C linkage was used as a criterion to measure the degree of tightness of the association between different contigs by standardizing the digestion sites of *Dpn*II on the genome sketch. The contigs were assembled into 46 pseudo-chromosomes using LACHESIS package tools. The Illumina paired-end reads were mapped to the assembled genome to assess assembly accuracy, resulting in a 98.81% mapping rate (Supplementary Table 3 and Figure 3B). The genome assembly captured 96.77% of the core eukaryotic genes from CEGMA18 (Supplementary Table 4) and 92.6% of the Embryophyta OrthoDB gene set in BUSCO19 (Supplementary Table 5), indicating a high level of completeness.

### Genome Annotation

We identified 1.56 Gb of non-redundant repetitive elements, representing approximately 55.85% of the *H. mutabilis* genome assembly. Because long terminal repeat retrotransposons (LTR-RTs) typically make a significant contribution to large genome size (Zhao and Ma, 2013), we estimated LTR-RT insertion time in *H. mutabilis*. We identified a round of LTR-RT burst approximately 2.5 million years ago (Mya), especially for the Ty3/Gypsy-del and Ty1/Copia-Retrofit families (Supplementary Table 6). The transposable elements (TEs) were primarily long terminal repeats (LTRs), which accounted for approximately 53% of the genome (Supplementary Figure 2).

We used *de novo* and homology-based gene prediction approaches and combined their results to annotate 118,222 protein-coding genes in the *H. mutabilis* genome. The average transcript length was 2,466.97 bp, with an average of 4.53 exons per gene and an average exon length of 218.86 bp. Compared with other model plants and Malvaceae species, the *H. mutabilis* genome contained a larger number of genes: *H. syriacus* (82,827 genes), *Arabidopsis thaliana* (26,869), *Theobroma cacao* (29,144), *G. raimondii* (35,526), *Corchorus capsularis* (29,356), *Herrania umbratica* (29,262), and *Durio zibethinus* (63,819) (Supplementary Table 7).

In addition to RNA-coding genes, we also identified 827 mature microRNAs (miRNAs), 3,604 transfer RNAs (tRNAs), 3,423 ribosomal RNAs (rRNAs), and 9,370 small nuclear RNAs (snRNAs) in the *H. mutabilis* genome (Supplementary Table 11).

The probable functions of the predicted genes were assessed by searching against public databases, including Swiss-Prot, NR, InterPro, and KEGG of 118,222 predicted genes in the *H. mutabilis* genome, 113,821 (96.3%) were assigned potential functions as a result of these database searches (Supplementary Table 8).

### Genome Evolution

**FIGURE 4:**
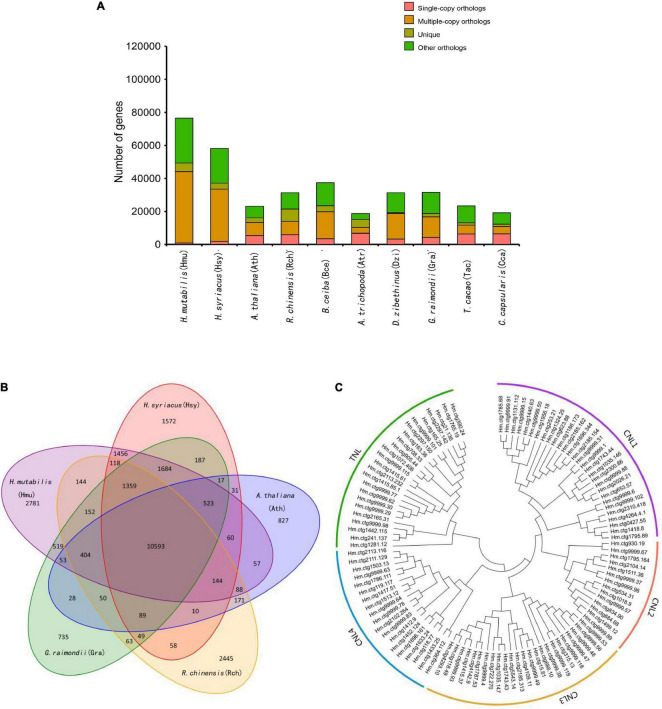
Comparative genomics and evolution of gene numbers. **(A)** The number of genes in cluster of ten plant species, showing a high gene number in *H. mutabilis* compared with the model plant *A. thaliana* and other angiosperm species. The number of multiple-copy paralogs is high in *H. mutabilis*. **(B)** Venn diagram showing the numbers of shared gene families among *H. mutabilis* (Hmu), *A. thaliana* (Ath), *H. syriacus* (Hsy), *R. chinensis* (Rch), and *G. raimondii* (Gra). **(C)** Phylogenetic relationships of NBS genes in *H. mutabilis*.

**FIGURE 5:**
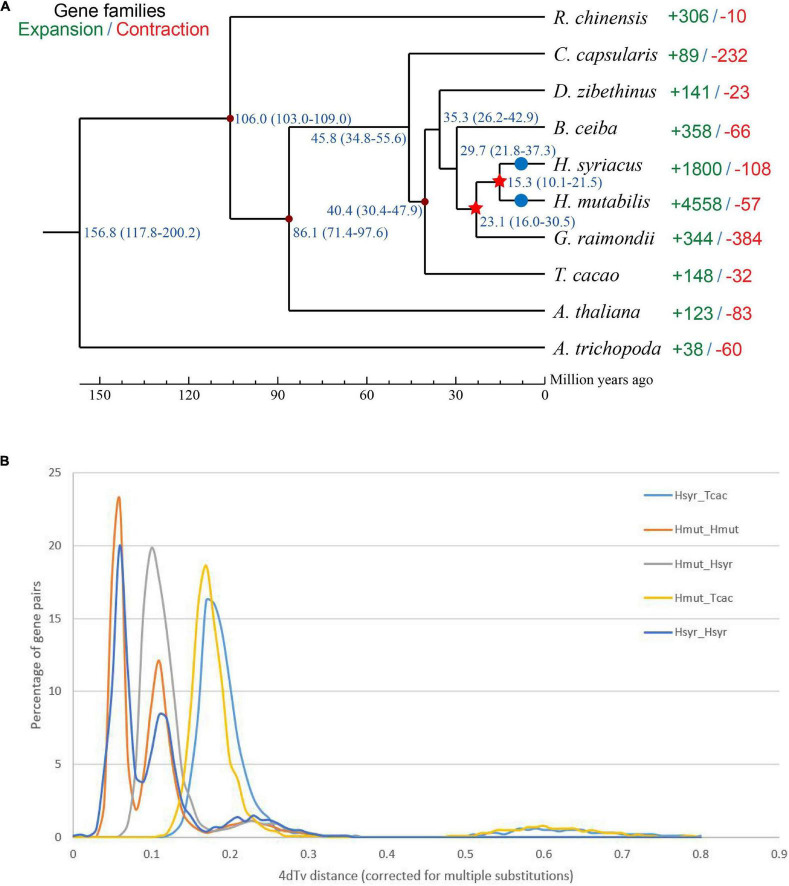
**(A)** Phylogenetic tree showing the close relationship between *H. mutabilis* and *H. syriacus*. Numbers outside the parentheses represent the divergence times of species (Mya), and numbers inside the parentheses indicate the confidence intervals. The red points indicated calibration points, and the red stars indicate WGD events and blue circles indicate diploidization events. **(B)** Distribution of 4Dtv distances.

Although morphological investigations have placed *H. mutabilis* in the Malvaceae family, there is still no phylogenomic analysis of its evolutionary position within the family based on whole-genome data. Here, we compared the *H. mutabilis* genome with the genome sequences of nine other angiosperm plants (*H. syriacus*, *A. thaliana*, *Rosa chinensis*, *Bombax ceiba*, *Amborella trichopoda*, *G. raimondii*, *T. cacao*, and *C. capsularis*). Orthologous protein groups were identified within the genomes, yielding a total of 30,208 gene families and 198 single-copy orthologs across ten species. There were 2,781 gene families specific to *H. mutabilis*, and 10,593 gene families were shared among all species investigated (Figure 4). We detected 4,558 gene families expansion when *H. mutabilis* and *H. syriacus* have diverged (Figure 5C).

We constructed a phylogenetic tree based on single-copy genes using PAML and estimated the divergence times among the 10 species. The Malvaceae family appeared to have diverged from a Tiliaceae–Malvaceae most recent common ancestor (MRCA) approximately 45.8 (34.8–55.6) Mya, and the *Hibiscus-Gossypium* divergence was estimated at 23.1 (16.0–30.5) Mya. *H. mutabilis* was most closely related to *H. syriacus*, with an estimated divergence time of approximately 15.3 (10.1–21.5) Mya (Figure 5A). In addition to the paleohexaploidization event shared by the eudicots, we observed three additional whole-genome duplication (WGD) events in *H. mutabilis*. *Hibiscus* and *Gossypium* shared a WGD event (13.3–20.0 Mya), *H. mutabilis* and *H. syriacus* shared a WGD event (10.76–21.51 Mya), and *H. mutabilis* and *H. syriacus* each underwent a WGD event (4.61–9.00 Mya) (Figure 5B).

### Flowering Time and Disease Resistance Genes

Genetic and molecular mechanisms of floral development are highly conserved among different plant species (Schiessl et al., 2014) and include four major flowering pathways that have been well characterized in *A. thaliana*. *H. mutabilis* is similar to *H. syriacus*, a short-day flowering plant with a long bloom period that produces more than 30 blossoms per day. Like those of *H. syriacus*, the flowers of *H. mutabilis* open daily and last for less than 48 h. Because flowering time is frequently dependent on gene copy number (Grover et al., 2015), we investigated the copy numbers of genes involved in the four major flowering pathways in *A. thaliana*, *T. cacao*, *G. raimondii*, *A. trichopoda*, *H. syriacus*, and *H. mutabilis*. Copy numbers of most flowering-related genes were higher in *H. mutabilis* than in other plants, including *T. cacao*, *G. raimondii*, *A. trichopoda*, and *H. syriacus*. In particular, the copy number of the plant-specific nuclear protein GIGANTEA (GI) was three to four times greater in *H. mutabilis* than in *A. trichopoda*, *T. cacao*, or *G. raimondii* (Table 1).

**TABLE 1:** Copy numbers of genes encoding flowering time regulators in five plant species.

Gene	*Arabidopsis* locus	Copy number				
		*H. mutabilis*	*H. syriacus*	*A. trichopoda*	*T. cacao*	*G. raimondii*
CO	AT5G15840	8	9	7	3	2
ELF4	AT2G40080	9	12	2	1	5
FCA	AT4G16280	4	0	1	2	1
FKE1	AT1G68050	0	3	1	1	2
FLK	AT3G04610	5	4	1	1	3
GI	AT1G22770	22	15	5	5	7
LFY	AT5G61850	4	4	1	1	1
LHY	AT1G01060	0	0	1	0	0
VIN3	AT5G57380	0	0	1	0	0
SOC1	AT2G45660	15	12	4	4	6
TFL	AT5G03840	24	13	6	5	7
SVP	AT1G24260	48	33	8	7	17
PHYA	AT1G09570	10	5	3	3	4
PHYB	AT2G18790	10	5	4	3	5
PHYC	AT5G35840	0	1	1	1	4
PHYE	AT4G18130	5	3	3	1	2

Nucleotide-binding site (NBS) and carboxy-terminal LRR domains are found in the majority of R proteins (DeYoung and Innes, 2006; Takken et al., 2006). Based on resistance domain analyses in the *H. mutabilis* genome, a total of 490 NBS-containing resistance genes were identified and classified into six groups: CC-NBS-LRR, CC-NBS, TIR-NBS-LRR, TIR-NBS, NBS-LRR, and NBS. In total, their gene numbers were approximately three times greater in *H. mutabilis* than in *A. thaliana* (170). This trend was particularly striking for the NBS genes, whose numbers were much higher in *H. mutabilis* (192 genes) than in *H. syriacus* (54), *T. cacao* (9), *G. raimondii* (4), and *A. thaliana* (3). Although *H. mutabilis* had the highest number of NBS-containing resistance genes among the five angiosperms (Table 2), its number of NBS-containing genes as a percentage of total genes was the lowest. All six NBS-containing groups existed in each plant genome, but their distributions differed among species.

**TABLE 2:** Numbers and classifications of genes encoding NBS-containing resistance proteins in five plant species.

Protein domain	Letter code	*H. mutabilis*	*H. syriacus*	*T. cacao*	*G. raimondii*	*A. thaliana*
CC-NBS-LRR	CNL	81	183	202	220	52
CC-NBS	CN	32	77	25	24	3
TIR-NBS-LRR	TNL	28	68	14	26	87
TIR-NBS	TN	10	9	3	1	17
NBS-LRR	NL	147	81	34	28	8
NBS	N	192	54	9	4	3
Total		490	472	287	303	170
% of total genes		0.41	0.53	0.97	0.81	0.63

### Transcriptome Sequencing Analysis

**FIGURE 6:**
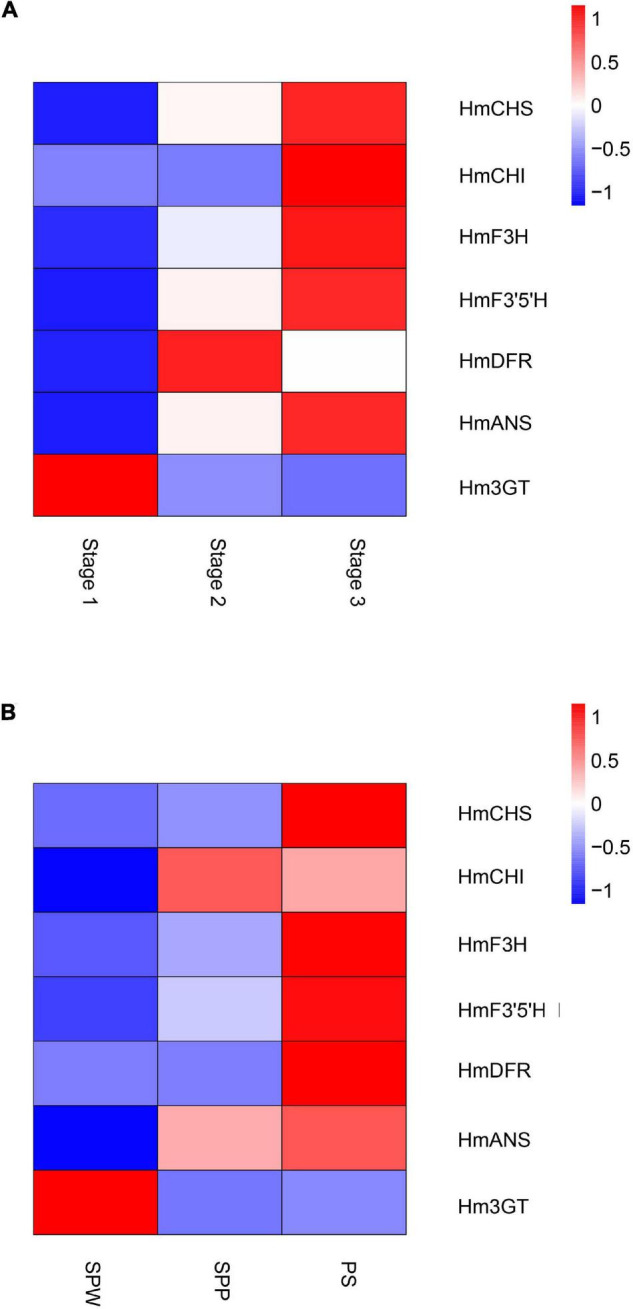
Heatmaps show the expression of key genes in the anthocyanin biosynthesis pathway of *H. mutabili*s. **(A)** Gene expression changes in flowers of *H. mutabilis* ‘Drunk girl’ as their color transitions from white to fully pink. **(B)** Gene expression in flowers of three different color cultivars: ‘single-petal white’ (SPW), ‘single-petal pink’ (SPP), and ‘Purple silk’ (PS).

Global gene expression patterns were quantified in three stages of the floral color transition of *H. mutabilis* ‘Drunk girl’: white (Stage 1), blended white and pink (Stage 2), and fully pink (Stage 3). A total of 9,492 genes were up-regulated from Stage 1 to Stage 2, and more than 15,000 genes were up-regulated from Stage 1 to Stage 3. A total of 8,481 genes were down-regulated from Stage 1 to Stage 2, and 15,839 genes were down-regulated from Stage 1 to Stage 3. In particular, we analyzed expression changes in anthocyanin-related genes at the three flower stages and present the results in a heatmap (Figure 6A). A number of key anthocyanin biosynthesis-related genes, such as Hmchs, HmchI, and Hmans, increased in expression from Stage 1 to Stage 3, consistent with the pattern of floral color development.

Key anthocyanin biosynthesis-related genes also differed in expression among different color cultivars of *H. mutabilis*, including ‘single-petal white,’ ‘single-petal pink,’ and ‘Purple silk.’ The highest expression levels were generally found in ‘Purple silk,’ which is a deep purple color form (Figure 6B).

## Discussion

Completeness and continuity are important indicators of genome assembly quality. In this study, we took advantage of the longer read lengths offered by ONT sequencing that have proven advantageous in the assembly of other plant genomes such as *Solanum pennellii* (Schmidt et al., 2017) and *Chrysanthemum nankingense* (Song et al., 2018). Here, we report the first genome data for *H. mutabilis* and estimate its genome size to be 2.68 Gb, far larger than that of *H. syriacus*. Our *H. mutabilis* genome assembled using Nanopore reads had a contig N50 of 2.02 Mb. We then used Hi-C data to cluster the contigs into forty-six chromosomes with a final scaffold N50 of 54.70 Mb. The genome contained complete copies of 92.6% of the BUSCO orthologs examined. This genome sequence will contribute to our understanding of the biosynthesis of natural products such as anthocyanins, although additional research is needed to directly link specific genes to individual traits. Nonetheless, our high-quality, annotated genome sequence provides insights into determinate flowering time and flower color in *H. mutabilis.*

Compared with the *H. syriacus* genome, the *H. mutabilis* genome was larger and contained more protein-coding genes. *H. mutabilis* and *H. syriacus* share an MRCA approximately 15.3 (10.1–21.5) Mya, and investigation of WGD timing in the *H. mutabilis* genome showed that two WGDs occurred after *H. mutabilis*–*H. syriacus* divergence and *H. mutabilis* speciation. WGD events and tandem duplications are the most important determinants of genome size variation in angiosperms (Piegu et al., 2006; El Baidouri and Panaud, 2013). This recent WGD event not only caused genome expansion in *H. mutabilis*, but may also have contributed to the morphological and physiological diversity of *H. mutabilis*. We inferred that gene losses, which had different frequencies in *H. mutabilis* and *H. syriacus*, made the *H. syriacus* genome smaller than that of *H. mutabilis*. *H. mutabilis* has a long bloom period and high blossom turnover. The copy numbers of most flowering-related genes, such as GI, CONSTANS (CO), and SUPPRESSOR OF OVEREXPRESSION OF CONSTANS1 (SOC1) were higher in *H. mutabilis* than in *T. cacao*, *A. thaliana*, and *H. syriacus*. These results show that *H. mutabilis* preserved many copies of flowering-related genes during the transition from a polyploid to a diploid genome.

The dynamic color change from white to pink in *H. mutabilis* ‘Drunk girl’ flowers is reported to be caused by variations in anthocyanin contents (Liu et al., 2008). Flavonoids are the major molecules involved in plant pigmentation (Lai et al., 2014) and include anthocyanins, flavan-3-ols (catechins and proanthocyanidins), flavanonols, flavonols, flavones, and phenolic acid (Lou et al., 2014). To date, regulation of the flavonoid pathway has been shown to occur primarily at the transcriptional level (Mol et al., 1998). Different species have distinct regulated genes, and these appear to be among the most important candidate genes for flower color determination (Casimiro-Soriguer et al., 2016; Jiao et al., 2020). To investigate the expression of anthocyanin-related genes over the course of flower development and in different color forms, we combined the high-quality genome sequence generated here with RNA-seq data from *H. mutabilis* The expression levels of anthocyanin biosynthetic genes such as Hmchs, HmchI, and Hmans were correlated and increased as flowers transitioned from white to pink. The pink flower color in cotton rose is related to the synthesis of cyanidin-based pigments (Chen et al., 2014), and our results indicate that low CHS, CHI, and ANS expression may inhibit cyanidin production in white flowers. Thus, combined genomic and transcriptomic analysis of *H. mutabilis* flowers indicated that structural genes had important roles in anthocyanin biosynthesis during the transition from white to pink flower coloration. In maize, an MYB-related protein and a bHLH containing protein interact to activate genes in the anthocyanin biosynthetic pathway (Schwinn et al., 2006). However, the functions of transcription factors, including MYBs, bHLHs, and WD40s, are unknown in *H. mutabilis*. The investigation of these TFs in cotton rose will be a subject for further research.

## Data Availability Statement

The datasets presented in this study can be found in online repositories. The names of the repository/repositories and accession number(s) can be found below: https://www.ncbi.nlm.nih.gov/genbank/, PRJNA717149.

## Author Contributions

YY and XLL: conceptualization. XG, MZ, and XDL: formal analysis. FL and ZZ: project administration. JM and XZ: resources. YY and XS: writing—original draft. XLL: writing—review and editing. All authors have read and agreed to the published version of the manuscript.

## Conflict of Interest

The authors declare that the research was conducted in the absence of any commercial or financial relationships that could be construed as a potential conflict of interest.

## Publisher’s Note

All claims expressed in this article are solely those of the authors and do not necessarily represent those of their affiliated organizations, or those of the publisher, the editors and the reviewers. Any product that may be evaluated in this article, or claim that may be made by its manufacturer, is not guaranteed or endorsed by the publisher.

## Footnotes

1
https://nanoporetech.com
