# Supplementary material for: A High-Quality, Chromosome-Level Genome Provides Insights Into Determinate Flowering Time and Color of Cotton Rose (Hibiscus mutabilis)
Source: Front Plant Sci. 2022 Feb 14;13:818206. doi: 10.3389/fpls.2022.818206 (PMC8896357; doi:10.3389/fpls.2022.818206)
Supplement: Supplementary file 1 [file Data_Sheet_1.docx]

Supplementary Material


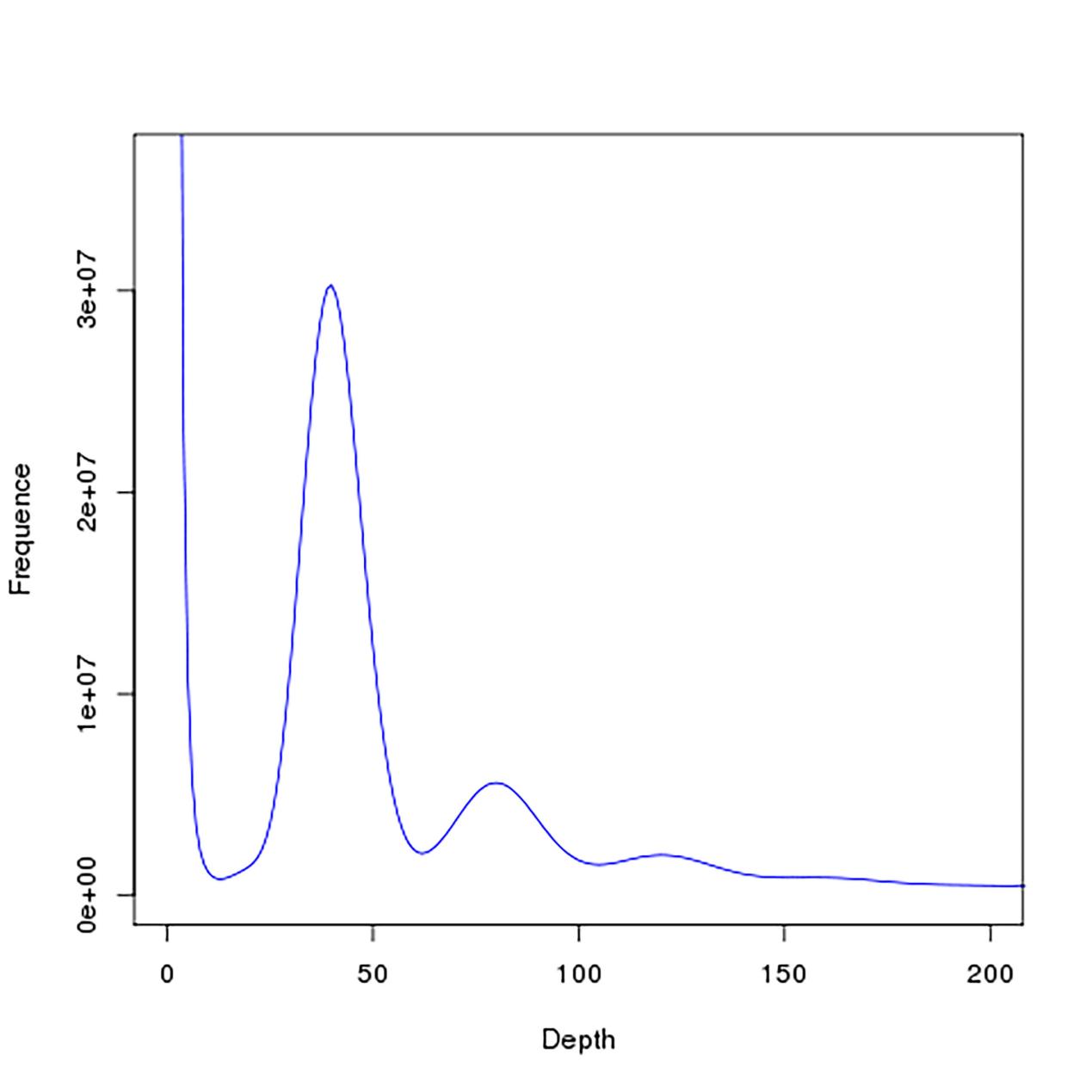


**Figure S1.** Distribution of 17-mer frequencies in the *Hibiscus mutabilis* genome.


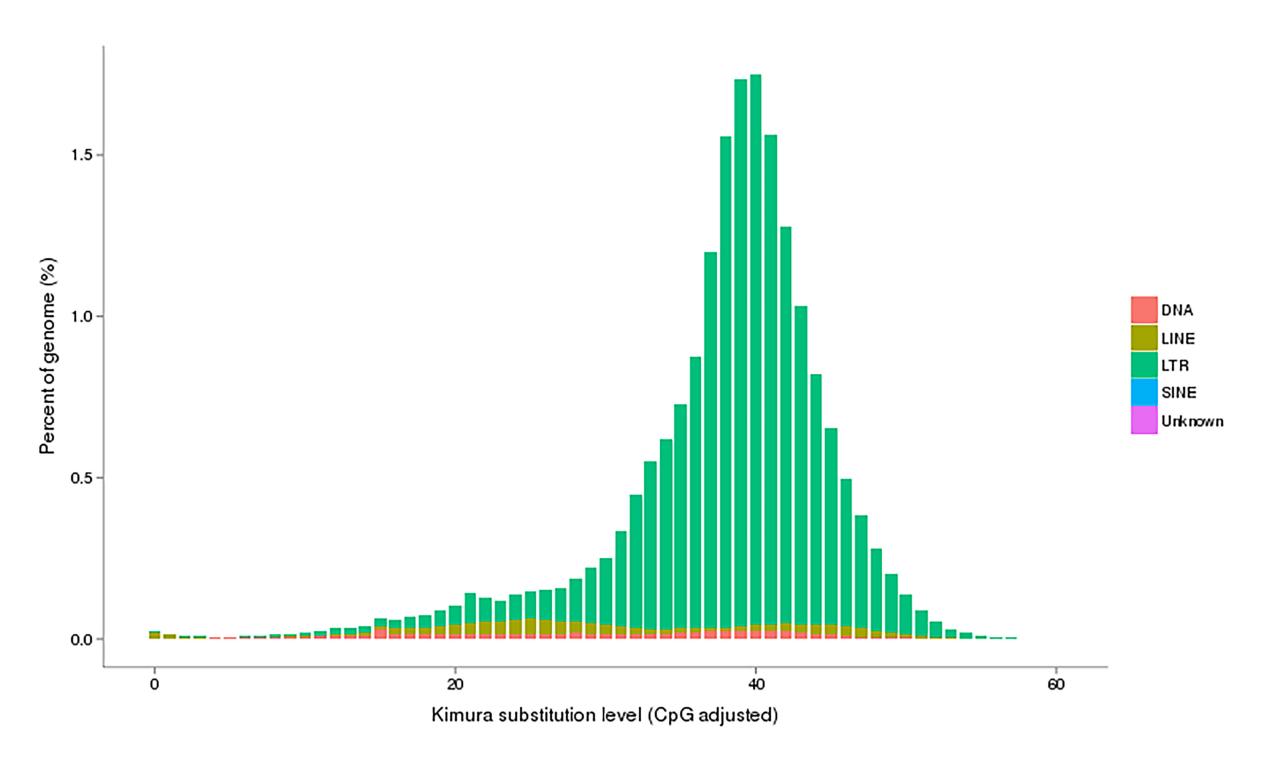


**Figure S2. Divergence distribution of transposable element (TE) families.** Red indicates DNA transposons (DNA), dark yellow indicates long interspersed nuclear elements (LINEs), green indicates long terminal repeats (LTRs), light blue indicates short interspersed nuclear elements (SINEs), and purple indicates unknown TEs.

| **Table S1. Statistics for K-mer analysis.** | | | | | | |
| --- | --- | --- | --- | --- | --- | --- |
| **Kmer** | **Depth** | **n_kmer** | **Genome size (M)** | **Revised Genome size (M)** | **Heterozygous rate (%)** | **Repeat rate (%)** |
| 17 | 40 | 122,166,178,364 | 3,054 | 3,032.98 | 0.09 | 77.73 |

| **Table S2. Quality assessment statistics of the assembled** Hibiscus mutabilis genome | | |
| --- | --- | --- |
| **Statistic** | **Contig length (bp)** | **Contig number** |
| N50 | 2,220,909 | 330 |
| Longest | 17,052,671 | 1 |
| Total | 2,676,003,569 | 5464 |
| Length > 2 kb | - | 5451 |

| **Table S3. Statistics of paired-end read mapping.** | | |
| --- | --- | --- |
|  | **Sample ID** | **Percentage** |
| Reads | Mapping rate (%) | 98.81 |
| Genome | Average sequencing depth | 95.92 |
|  | Coverage (%) | 97.93 |
|  | Coverage at least 4X (%) | 97.63 |
|  | Coverage at least 10X (%) | 97.35 |
|  | Coverage at least 20X (%) | 97.00 |

| **Table S4. CEGMA results for the** Hibiscus mutabilis genome. | | | | |
| --- | --- | --- | --- | --- |
| **Species** | **Complete** | | **Complete + partial** | |
|  | **Protein Number** | **% completeness** | **Protein Number** | **% completeness** |
| *Hibiscus mutabilis* | 237 | 95.56 | 240 | 96.77 |
| A protein is classified as complete if the alignment of the predicted protein to the HMM profile represents at least 70% of the original KOG domain; otherwise it is classified as partial. | | | | |

| **Table S5. BUSCO (Benchmarking Universal Single-Copy Orthologs) results for the** Hibiscus mutabilis **genome.** | |
| --- | --- |
| **Species** | **BUSCO notation assessment results** |
| *Hibiscus mutabilis* | C 92.6% [S 36.0%, D 56.6%], F 1.5%, M 5.9%, n 1440 |
| C, Complete BUSCOs. S, Complete and single-copy BUSCOs. D, Complete and duplicated BUSCOs. F, Fragmented BUSCOs. M, Missing BUSCOs. n, Total BUSCO groups searched. | |

| **Table S6. Categories of TEs predicted in the *Hibiscus mutabilis* genome.** | | | | | | | |
| --- | --- | --- | --- | --- | --- | --- | --- |
|  |  | **Repbase + De novo** | | **TE Proteins** | | **Combined TEs** | |
|  |  | **Length (bp)** | **% in Genome** | **Length (bp)** | **% in Genome** | **Length (bp)** | **% in Genome** |
| DNA transposon | DNA | 21,123,777 | 0.79 | 19,103,585 | 0.71 | 37,038,850 | 1.38 |
| Retrotransposon | LINE | 24,769,782 | 0.93 | 40,812,681 | 1.53 | 62,060,731 | 2.32 |
|  | SINE | 824,053 | 0.03 | 0 | 0 | 824,053 | 0.03 |
|  | LTR | 1,365,856,884 | 51.04 | 487,789,567 | 18.23 | 1,422,404,552 | 53.15 |
|  | Unknown | 13,718,932 | 0.51 | 0 | 0 | 13,718,932 | 0.51 |
|  | Total | 1,420,759,266 | 53.09 | 546,018,624 | 20.4 | 1,523,904,923 | 56.95 |
| Note: "Unknown" refers to repeats that could not be classified by RepeatMasker. | | | | | | | |

| **Table S7. General statistics of predicted protein-coding genes.** | | | | | | | | | | | | | |  |
| --- | --- | --- | --- | --- | --- | --- | --- | --- | --- | --- | --- | --- | --- | --- |
| **Gene set** | | **Number** | **Average transcript length (bp)** | | **Average CDS length (bp)** | **Average exon length (bp)** | | | **Average intron length (bp)** | | | **Average exons per gene** | |  |
| De novo# | Augustus | 153,217 | 1,919.29 | | 874.40 | 3.89 | | | 224.95 | | | 361.91 | |  |
|  | GlimmerHMM | 288,025 | 7,505.89 | | 535.51 | 2.70 | | | 198.58 | | | 4108.17 | |  |
|  | SNAP | 291,826 | 2,710.49 | | 550.67 | 3.36 | | | 163.96 | | | 915.74 | |  |
|  | GeneID | 215,422 | 4,116.11 | | 675.42 | 3.83 | | | 176.37 | | | 1215.95 | |  |
|  | Genscan | 161,173 | 9,953.25 | | 951.84 | 5.17 | | | 184.17 | | | 2159.51 | |  |
| Homolog$ | *Arabidopsis thaliana* | 120,910 | 1,849.61 | | 877.86 | 3.65 | | | 240.58 | | | 366.85 | |  |
|  | *Corchorus capsularis* | 87,464 | 2,753.45 | | 1,154.87 | 4.36 | | | 264.70 | | | 475.36 | |  |
|  | *Durio zibethinus* | 159,657 | 1,789.15 | | 861.70 | 3.40 | | | 253.71 | | | 387.02 | |  |
|  | *Gossypium raimondii* | 110,738 | 2,366.26 | | 1,048.62 | 4.05 | | | 259.05 | | | 432.30 | |  |
|  | *Herrania umbratica* | 180,085 | 1,911.61 | | 843.96 | 3.31 | | | 255.31 | | | 463.05 | |  |
|  | *Hibiscus syriacus* | 70,336 | 2,598.97 | | 1,103.22 | 4.45 | | | 248.11 | | | 433.99 | |  |
|  | *Theobroma cacao* | 120,357 | 2,241.01 | | 993.45 | 3.80 | | | 261.44 | | | 445.56 | |  |
| RNA_Seq | PASA | 107,159 | 1,915.71 | | 847.78 | 3.92 | | | 216.51 | | | 366.27 | |  |
|  | Cufflinks | 136,661 | 3,796.65 | | 1,750.59 | 5.86 | | | 298.50 | | | 420.61 | |  |
| EVM | | 167,861 | 2,065.69 | | 835.31 | 3.86 | | | 216.60 | | | 430.73 | |  |
| Pasa-update* | | 167,633 | 2,043.10 | | 834.11 | 3.84 | | | 217.48 | | | 426.39 | |  |
| Final set* | | 118,222 | 2,466.97 | | 992.41 | 4.53 | | | 218.86 | | | 417.19 | |  |
| #Statistics calculated from the gene set predicted by each method. | | |  |  | | |  |  | |  |  | |  | |
| $Statistics calculated from the gene set predicted using homologous proteins from each species. | | |  |  | | |  |  | |  |  | |  | |
| *Statistics calculated based on the Medicago ruthenica genome. |  | | |  | | |  |  | |  |  | |  | |

| **Table S8. General statistics of mapping rates to functional databases of protein-coding genes.** | | | |
| --- | --- | --- | --- |
| **Database** | | **Number Annotated** | **Percent Annotated (%)** |
| NR | | 113,709 | 96.2 |
| Swiss-Prot | | 89,978 | 76.1 |
| KEGG | | 84,397 | 71.4 |
| InterPro | All | 94,421 | 79.9 |
|  | Pfam | 86,066 | 72.8 |
|  | GO | 64,120 | 54.2 |
| Annotated | | 113,821 | 96.3 |
| Total | | 118,222 | - |

| **Table S9. Program tools** | | |
| --- | --- | --- |
| Name | Version | Parameters |
| wtdbg2 | 2.5 | max_depth = 50；node-drop=0.25；node-len=1536,2048,2560,3072,3548； node-max=400；brute_force=1；kmer=21 |
| LACHESIS | 201701 | CLUSTER_N = 46;CLUSTER_CONTIGS_WITH_CENS = -1;CLUSTER_MIN_RE_SITES = 927; CLUSTER_MAX_LINK_DENSITY = 9;CLUSTER_NONINFORMATIVE_RATIO = 0;CLUSTER_DRAW_HEATMAP = 1;CLUSTER_DRAW_DOTPLOT = 1 |
| racon | v1.3.1 | default |
| pilon | 1.22 | default |

**Table S10. Summary statistics demonstrating the high quality of the Hi-C map of H. mutabilis.**

| **Category** | **Length** | | **Number** | |
| --- | --- | --- | --- | --- |
|  | **Contigs (bp)** | **Scaffolds (>100 bp)** | **Contigs** | **Scaffolds (>100 bp)** |
| Total | 2,676,003,569 | 2,676,237,573 | 5,598 | 3,254 |
| Max | 17,052,671 | 89,504,759 | - | - |
| Number ≥2000 | - | - | 5,585 | 3,241 |
| N50 | 2,021,431 | 54,699,351 | 354 | 20 |

**Table S11. General statistics for non-coding RNAs in the H. mutabilis genome.**

| **Type** | **Number** | **Total**  **length (bp)** | **Average**  **length (bp)** | **Percentage of**  **genome (%)** |
| --- | --- | --- | --- | --- |
| miRNA | 827 | 103,898 | 125.63 | 0.00388 |
| tRNA | 3,604 | 270,734 | 75.12 | 0.01012 |
| rRNA | 3,423 | 520,604 | 152.09 | 0.01946 |
| snRNA | 9,370 | 1,020,234 | 108.88 | 0.03813 |

**Table S12. Genes used for gene family clustering in each species**

| **Species** | **Genes Number** |
| --- | --- |
| *H.mutabilis* | 118,222 |
| *H.syriacus* | 82,773 |
| *G.raimondii* | 35,226 |
